
JOURNAL INFORMATION
==============================
NLM Title Abbreviation: ScientificWorldJournal
Journal ID: ScientificWorldJournal
NLM Journal ID: 101131163
Journal ID: TSWJ

The Scientific World Journal

ISSN: 2356-6140
EISSN: 1537-744X
Publisher: Wiley

ARTICLE INFORMATION
==============================
PMCID: PMC5848727
PMID: 18335149
DOI: 10.1100/tsw.2008.44
Article ID: 361828
Article version: 1
Subjects: Clinical Image

Essential Type II Mixed Cryoglobulinemia Causing Pyoderma Gangrenosum–Like Ulcers

Namazi M. R. 1 2 *
Kerchner K. R. 1
Pichardo R. O. 1
1 Dermatology Department, Wake-Forest University, Baptist Medical Center, North Carolina, USA
2 Dermatology Department, Shiraz University of Medical Sciences, Shiraz, Iran
*M. R. Namazi: namazi_mr@yahoo.comAcademic Editor: Daniel Batlle

Publication date: 2008
Electronic publication date: 2008 Feb 25
Volume: 8
First page: 228
Last page: 228
Received 2008 Jan 29; Revised 2008 Jan 29; Accepted 2008 Feb 4
Copyright: Copyright © 2008 M. R. Namazi et al.
Copyright year: 2008
License: This is an open access article distributed under the Creative Commons Attribution License, which permits unrestricted use, distribution, and reproduction in any medium, provided the original work is properly cited.
License URL: https://creativecommons.org/licenses/by/3.0/

==============================
Clinical Image
*TheScientificWorldJOURNAL* (2008) 8, 228
ISSN 1537-744X; DOI 10.1100/tsw.2008.44

### Essential Type II Mixed Cryoglobulinemia Causing Pyoderma Gangrenosum–Like Ulcers

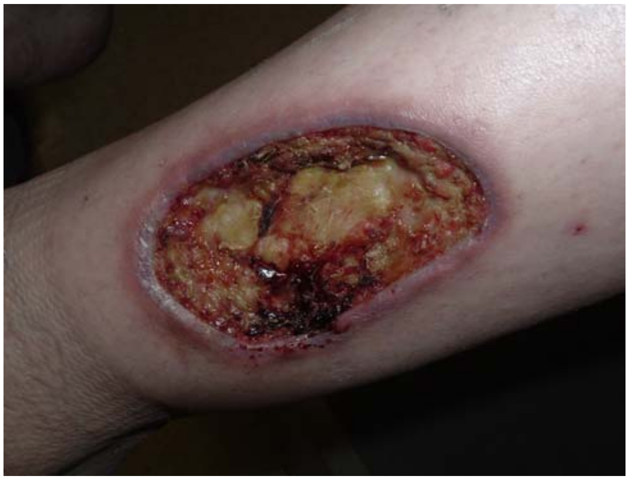

**Figure.** Pyoderma gangrenosum–like lesions due to cryoglobulinemia.

M.R. Namazi[1,2,]*, K.R. Kerchner[1], and R.O. Pichardo[1]

*[1]Dermatology Department, Wake-Forest University Baptist Medical Center, North Carolina; [2]Dermatology Department, Shiraz University of Medical Sciences, Shiraz, Iran*

**E-mail:** namazi_mr@yahoo.com

*Received January 29, 2008; Revised January 29, 2008; Accepted February 4, 2008; Published February 25, 2008*

A 47-year-old, otherwise healthy woman presented with multiple deep ulcers located primarily on her lower extremities that presented 6 days ago as blisters. A biopsy revealed livedoid vasculopathy, without any evidence of vasculitis. Extensive laboratory workup was positive only for type II cryoglobulins.

Type II cryoglobulinemia is caused by monoclonal rheumatoid factors. Cutaneous manifestations are nearly always present in cryoglobulinemia. Lesions have a predilection for dependent areas and include erythematous macules and purpuric papules (90–95%), as well as ulcerations (10–25%). Livedo reticularis, Raynaud phenomenon, and nailfold capillary abnormalities are also noted. Presentation with deep ulcers may lead to a misdiagnosis of pyoderma gangrenosum.

**This article should be cited as follows:**

Namazi, M.R., Kerchner, K.R., and Pichardo, R.O. (2008) Essential type II mixed cryoglobulinemia causing pyoderma gangrenosum–like ulcers. *TheScientificWorldJOURNAL* **8**, 228. DOI 10.1100/tsw.2008.44.

*Corresponding author.
©2008 with author.
Published by TheScientificWorld; www.thescientificworld.com
